# Enterovirus Detection Trends Based on Respiratory Specimens from a Single Tertiary Hospital in Korea (2018–2024): A Retrospective Study Using Multiplex PCR Data

**DOI:** 10.3390/v17070991

**Published:** 2025-07-16

**Authors:** Jeong Su Han, Sung Hun Jang, Jae-Sik Jeon, Jae Kyung Kim

**Affiliations:** 1 Department of Biomedical Laboratory Science, College of Health Sciences, Dankook University, Cheonan-si 31116, Republic of Korea; jshan1162@naver.com (J.S.H.); zenty87@naver.com (J.-S.J.); 2 Department of Medical Laser, Graduate School of Medicine, Dankook University, Cheonan-si 31116, Republic of Korea; well8143@naver.com

**Keywords:** enterovirus, epidemiology, respiratory viruses, multiplex PCR, pediatrics, COVID-19

## Abstract

Enteroviruses (EVs) cause broad clinical manifestations, particularly in children. Certain serotypes have been implicated in respiratory infections; however, epidemiological studies analyzing EV circulation based on clinical respiratory specimens are limited in Korea. This retrospective study evaluates EV detection patterns in respiratory specimens to demonstrate their clinical and epidemiological significance as respiratory pathogens in Korea. Respiratory samples collected from outpatient and hospitalized patients with respiratory symptoms at Dankook University Hospital between 2018 and 2024 were analyzed. EV detection patterns were analyzed by year, season, sex, and age. EVs were detected in 303/6292 respiratory specimens. The highest and lowest positivity rates were in 2018 (8.2%) and 2020 (1.6%), likely due to non-pharmaceutical interventions during the COVID-19 pandemic. The highest positivity rates were in summer and autumn, and in children aged 2–11 years and infants aged 0–1 years. EV positivity did not differ significantly between sexes. Significant differences were identified across years, seasons, and age groups. EVs can be detected in respiratory specimens from symptomatic patients and exhibit a marked seasonal distribution and elevated positivity rates in pediatric populations. Hence, EVs may act as atypical respiratory pathogens, underscoring the need for integrated public health surveillance and seasonal prevention strategies.

## 1. Introduction

Enteroviruses (EVs) are positive-sense single-stranded RNA viruses within the *Picornaviridae* family that are recognized as etiologic agents of various clinical conditions in children. These include meningitis, hand-foot-and-mouth disease, and myocarditis [1]. Historically, EVs have been closely linked with gastrointestinal tract and central nervous system infections. However, recent studies have identified specific serotypes, including Enterovirus D68 (EV-D68), as emerging respiratory pathogens capable of infecting the upper and lower respiratory tracts [2]. EV-D68 has been implicated in severe asthma exacerbations, respiratory failure, and neurologic complications such as acute flaccid myelitis, expanding the clinical relevance of EVs beyond the gastrointestinal system and positioning them as atypical respiratory viruses [3]. EVs are classified into four species (A to D) with distinct seasonal epidemic patterns, predominantly affecting pediatric populations during the summer and early autumn [4].

Advancements in molecular diagnostics have led to the detection of EVs in multiplex respiratory virus panels, often alongside rhinoviruses, complicating clinical interpretation and epidemiological surveillance [5]. Accurate clinical diagnosis and effective public health interventions require enhanced knowledge and monitoring of EV respiratory infections. Meanwhile, a paucity of data exists regarding the detection rate and epidemiological characteristics of EV infection, including age-specific and seasonal distribution, based on respiratory specimens. Most studies employ traditional samples, such as stool or cerebrospinal fluid, with limited retrospective analyses based on respiratory samples in real-world clinical settings [6].

The coronavirus disease-2019 (COVID-19) pandemic markedly altered the transmission dynamics of respiratory viruses. Non-pharmaceutical interventions (NPIs), including social distancing, mask-wearing, and mobility restrictions, suppressed the global circulation of enveloped viruses like influenza and respiratory syncytial virus (RSV) [7]. Meanwhile, non-enveloped viruses, including rhinoviruses, adenoviruses, and EVs, persisted, highlighting their resilience and the need for continued surveillance [8].

The current study employs multiplex PCR to evaluate the annual, monthly, seasonal, and sex- and age-specific trends in EV detection using respiratory specimens from patients presenting with respiratory symptoms at a tertiary care center from 2018 to 2024 in Republic of Korea. The primary objective is to demonstrate the clinical and epidemiological significance of EVs as respiratory pathogens, rather than solely gastrointestinal agents. The findings of this study offer foundational data to improve EV surveillance, identify high-risk pediatric populations, and establish seasonally tailored prevention strategies [9].

## 2. Materials and Methods

### 2.1. Study Design

This retrospective descriptive study examines respiratory virus genetic testing results for respiratory samples collected from June 2018 to December 2024 at the Department of Laboratory Medicine, Dankook University Hospital, Cheonan, Republic of Korea. A total of 6292 patients (4003 males and 2289 females) were included. Respiratory virus diagnostic testing was performed on all patients presenting with respiratory symptoms (e.g., cough, sputum production, dyspnea) in outpatient or inpatient settings. Respiratory specimens (e.g., nasopharyngeal swabs, sputum, bronchoalveolar lavage fluid) were collected based on clinical judgment, regardless of age, sex, or hospitalization status.

### 2.2. PCR Testing and Enterovirus Detection

After collection, samples were tested immediately using the AdvanSure RV Real-Time PCR kit (LG Chem, Cheongju, Republic of Korea) and interpreted per the manufacturer’s guidelines. Only EV results were extracted and analyzed. EV detection was categorized dichotomously as “Positive” or “Negative” according to the test reports. Cases with co-detection of other viral pathogens were excluded from analysis. All PCR assays were conducted in the molecular diagnostics laboratory of the Department of Laboratory Medicine at Dankook University Hospital, following standardized internal standard operating procedures (SOPs).

### 2.3. Data Collection

De-identified data were collected, including patient age, sex, testing date, and EV test results. All data were extracted from the hospital’s Laboratory Information System (LIS), and descriptive statistics were applied to analyze EV detection trends. Analyses included annual detection counts and positivity rates; seasonal distribution, categorized as spring (March–May), summer (June–August), autumn (September–November), and winter (December–February); and distribution by sex and age groups. Age stratification was based on the International Council for Harmonisation of Technical Requirements for Pharmaceuticals for Human Use (ICH) E11 guidelines: infants (0–1 years), children (2–11 years), adolescents (12–18 years), adults (19–64 years), and older adults (≥65 years).

### 2.4. Statistical Analysis

All statistical analyses were performed using SPSS software (version 17.0; SPSS Inc., Chicago, IL, USA) to assess the temporal trends in EV infections and compare positivity rates by age group, sex, and season. The chi-square (χ^2^) test (two-tailed) was used to evaluate differences between subgroups. A *p*-value < 0.05 was considered statistically significant.

## 3. Results

### 3.1. Annual Trends

**Figure 1:**
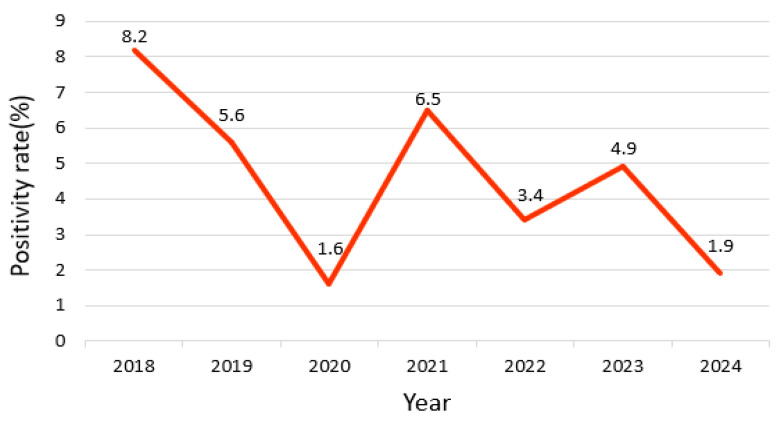
Annual positivity rate of enterovirus detected in respiratory specimens, 2018–2024.

Between January 2018 and December 2024, 303 cases at Dankook University Hospital tested positive for EV via multiplex PCR among patients with respiratory symptoms. EV detection was highest in 2018 (*n* = 76, 8.2%) and 2019 (*n* = 81, 5.6%), indicating relatively high detection levels before the COVID-19 pandemic. However, in 2020, positive cases declined sharply to 13 (1.6%). Although a rebound occurred in 2021 (40 cases, 6.5%), numbers fluctuated thereafter: 30 cases (3.4%) in 2022, 50 (4.9%) in 2023, and 13 (1.9%) in 2024. The annual variation was statistically significant (χ^2^ = 61.2, degrees of freedom [df] = 6, *p* < 0.001; Figure 1, Table 1), suggesting that annual fluctuations in EV circulation reflect true epidemiological changes rather than random variation, and EV activity has not fully recovered to its pre-pandemic patterns.

### 3.2. Monthly and Seasonal Distribution

**Figure 2:**
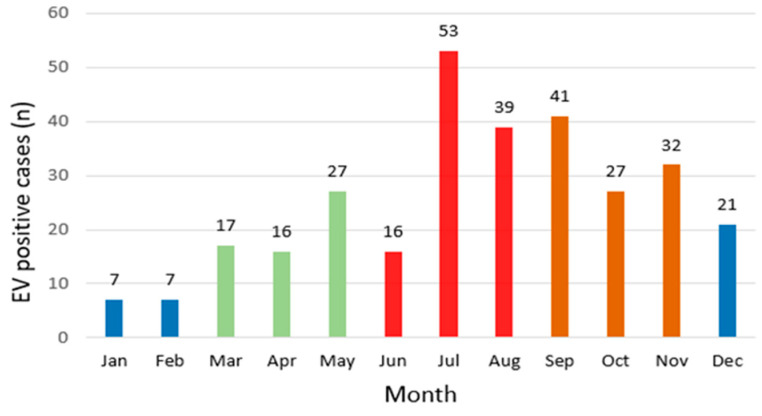
Monthly distribution of enterovirus (EV)-positive cases, 2018–2024. Bars are color-coded by season: spring (March–May, green), summer (June–August, red), autumn (September–November, brown), and winter (December–February, blue).

EV-positive cases exhibited a typical seasonal distribution, primarily occurring in summer (June–August) and autumn (September–November). Of the 303 positive cases, 108 (35.6%) occurred in summer and 100 (33.0%) in autumn. In contrast, spring (March–May) and winter (December–February) accounted for 60 (19.8%) and 35 (11.5%) cases, respectively. When considering the number of tests performed, the seasonal positivity rates were 6.6% in summer (108/1620), 6.0% in autumn (100/1644), 4.6% in spring (60/1298), and 2.0% in winter (35/1730), representing a statistically significant difference across seasons (χ^2^ = 47.2, df = 3, *p* < 0.001; Table 2). The monthly distribution of EV-positive cases peaked in July, confirming summer predominance (Figure 2). Before the pandemic, EV activity was concentrated in summer (2018), extending into summer and autumn in 2019. However, this seasonal pattern shifted during the pandemic period. In 2021, most positive cases were concentrated between September and November, indicating an atypical distribution. Furthermore, in 2023 and 2024, positive cases became increasingly concentrated in spring (March–May).

### 3.3. Distribution by Sex

**Figure 3:**
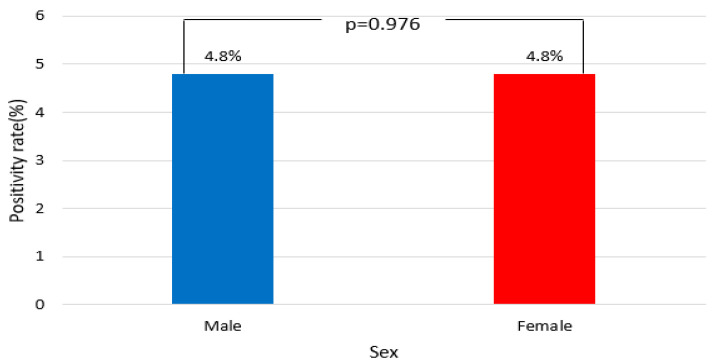
Enterovirus (EV) positivity rates by sex, 2018–2024.

From 2018 to 2024, the EV positivity rate was identical for both sexes, with a rate of 4.8%. Among 4003 specimens from males, 193 tested positive (4.8%), while among 2289 female specimens, 110 were positive (4.8%) (Figure 3). The observed and expected values showed minimal deviation, with no statistically significant differences between males and females (χ^2^ = 0.001, df = 1, *p* = 0.976; Table 3).

### 3.4. Age and Demographic Characteristics

**Figure 4:**
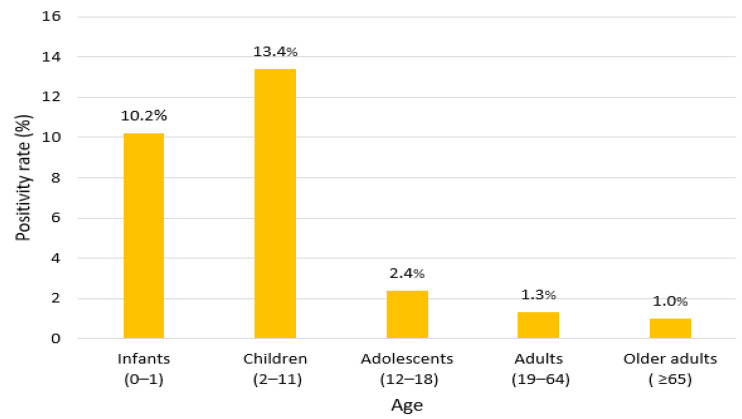
Age-specific enterovirus (EV) positivity rates, 2018–2024. Numbers above each bar indicate positivity rates (%).

The median age of patients who tested positive for EV was 6.5 years, with an interquartile range (IQR) of 1–5.7 years. Children under the age of 5 accounted for 67.7% of all EV-positive cases, with the highest proportion in the 1–4 years age group (*n* = 132, 43.6%). Age group analysis revealed the highest positivity rate in children aged 2–11 years (13.4%, 123/917), followed by infants aged 0–1 years (10.2%, 131/1276; Figure 4). The positivity rate for adolescents (12–18 years) was 2.4% (4/162), that for adults (19–64 years) was 1.3% (18/1314), and that for for older adults (≥65 years) was 1.0% (27/2623; Table 4). The differences across age groups were statistically significant (χ^2^ = 348.5, df = 4, *p* < 0.001).

## 4. Discussion

This study provides a quantitative, long-term (2018–2024) analysis of EV detection patterns based on respiratory specimens collected from 6292 patients with respiratory symptoms at a tertiary hospital in Korea. Using multiplex PCR, it investigates EV seasonality, demographic distribution, and annual trends, highlighting the under-recognized role of EVs in respiratory infections and epidemiological shifts associated with the COVID-19 pandemic.

Annual trend analysis revealed high EV positivity rates in 2018 (8.2%) and 2019 (5.6%), followed by a sharp decline in 2020 (1.6%) during the COVID-19 pandemic. This reflects the suppressive effect of NPIs like social distancing and mask-wearing on enveloped respiratory virus transmission [10,11]. Meanwhile, non-enveloped viruses, including EVs, rhinoviruses, and adenoviruses, persisted during the pandemic, suggesting structural resilience [12,13]. Compared to enveloped viruses such as influenza and RSV, non-enveloped viruses including EVs demonstrated greater environmental stability and resistance to disinfection measures, which may explain their relative persistence during the pandemic despite stringent NPIs [14,15]. Since 2021, a gradual resurgence has been observed in EV activity, peaking at 4.9% in 2023, potentially due to relaxed NPIs and resumed social activities [16]. These post-pandemic surges were similarly reported by the Centers for Disease Control and Prevention and European health agencies [17,18]. In particular, the increased detection of EV-D68, responsible for causing severe respiratory illness—including increased intensive care unit (ICU) admissions, respiratory failure, and acute flaccid myelitis—warrants genotypic surveillance of circulating EV strains [19,20].

Seasonally, EV positivity peaked during summer (6.6%) and autumn (6.0%), consistent with established circulation patterns in temperate regions, such as China and Europe [4,21,22]. These findings align with the known seasonality of EVs, which tend to circulate more actively during warm and humid months [23]. However, deviations from this pattern were noted: in 2021, cases clustered in autumn (September–November), while in 2023 and 2024, peak activity shifted to spring (March–May). These changes suggest that the altered public health environment during and after the pandemic may have structurally influenced the seasonal dynamics of EV transmission. Ongoing surveillance is necessary to detect atypical shifts and provide early warnings of unseasonal outbreaks.

Age-specific analysis revealed a median age of 6.5 years among EV-positive cases, with 68.9% detected in children under 5 [24,25]. Children aged 2–11 years had the highest positivity rates (13.4%), followed by infants aged 0–1 years (10.2%), while adults and older adults had significantly lower rates. These findings align with previous studies, reflecting higher susceptibility among younger populations attributed to immature immunity and close contact in daycare settings [26]. Nevertheless, 14.8% of EV-positive cases occurred in adults, indicating that EV infections are not exclusive to children. Specifically, immunocompromised or chronically ill adults are at a greater risk of severe outcomes from EV [27]. In contrast, sex-based analysis showed equal positivity rates for males and females, suggesting that EV transmission is influenced more by environmental exposure and host immunity than biological sex [28].

This study also has certain strengths. First, it analyzes EV trends based on respiratory specimens collected clinically over multiple years, offering a new perspective compared to conventional gastrointestinal-focused surveillance systems [29]. Second, it includes data spanning the COVID-19 pandemic, providing dynamic insights into EV epidemiology that may facilitate the early detection of atypical outbreak patterns. Third, due to the scarcity of studies with a similar scale and methodologies, the findings of the current study address a gap in the literature and support the expansion and refinement of EV surveillance systems.

Nevertheless, some limitations must also be acknowledged. First, as a single-center retrospective study, the findings may not fully represent regional or national trends. However, the use of standardized molecular diagnostics (SOP-based multiplex PCR) partially supports the data’s generalizability. Second, genotypic or serotypic information was not included, limiting the assessment of specific EV strains such as EV-D68 and their correlation with clinical severity. Third, test-level data prevents analysis of individual clinical outcomes, hospitalization status, or complications. Finally, co-detection of other respiratory viruses was excluded from the dataset, and the interaction effects between age, sex, and seasonality were not statistically modeled.

Future studies should incorporate genotyping or serotyping, evaluate co-infections, and link clinical outcome data in a multicenter prospective cohort design. Applying multivariate regression models with interaction terms (e.g., age × season, sex × age) will allow for more precise identification of risk factors and inform targeted public health responses. The proposed surveillance framework can be adapted to detect temporal and seasonal shifts in EV activity. Expanded early screening and strategic adjustment of target populations may be required in cases where epidemic peaks are delayed to late autumn or shift to early spring.

## 5. Conclusions

This study quantitatively analyzed 6292 respiratory specimens from a tertiary care hospital between 2018 and 2024 to investigate the long-term epidemiological trends, seasonality, and demographic characteristics of EV infections. EV infections were predominantly observed in pediatric populations and exhibited a distinct seasonal pattern, with peak activity in summer and autumn. Differences in EV circulation before and after the COVID-19 pandemic, along with the emergence of atypical seasonal patterns, suggest that NPIs and broader societal changes influenced EV epidemiology. These findings emphasize the role of EVs in respiratory infections and suggest reconsidering their inclusion in national respiratory virus surveillance systems.

Such adaptive strategies can enable earlier detection of atypical EV outbreaks, reduce resource utilization, and improve public health system efficiency. In summary, this long-term surveillance provides valuable insights into the age- and season-specific dynamics of enterovirus infections in Korea. These findings can inform clinical suspicion and laboratory testing strategies across different patient populations, highlighting the importance of timely diagnosis and targeted clinical management. Moreover, the observed epidemiological patterns can serve as a foundation for future public health measures, including the targeted design of prevention strategies, development and evaluation of vaccines, and optimization of national surveillance and early warning systems for enterovirus outbreaks. By leveraging these data, it may be possible to reduce the disease burden associated with enterovirus infections and better protect vulnerable populations, especially young children, from epidemic and sporadic outbreaks. Additionally, integrating multicenter studies with genotyping, co-infection assessment, and clinical outcome analysis will be essential for quantifying the disease burden of EV infections and supporting the development of targeted public health guidelines.

## Figures and Tables

**Table 1:** Annual distribution of enterovirus test results and positivity rates with observed and expected values, 2018–2024.

Year	Total	Positive (Observed)	Positive (Expected)	Negative (Observed)	Negative (Expected)	Positivity Rate (%)	χ^2^	*p*
2018	926	76	44.4	850	881.4	8.2	61.2	<0.001
2019	1432	81	68.9	1351	1363	5.6		
2020	792	13	38.1	779	753.8	1.6		
2021	613	40	29.5	573	583.4	6.5		
2022	860	30	41.4	830	818.5	3.4		
2023	1016	50	48.9	966	967	4.9		
2024	653	13	31.4	640	621.5	1.9		

Overall chi-square (χ^2^) and *p* values are presented.

**Table 2:** Seasonal distribution of enterovirus positivity with observed and expected frequencies.

Season	Total	Positive (Observed)	Positive (Expected)	Negative (Observed)	Negative (Expected)	Positivity Rate (%)	χ^2^	*p*
Spring	1298	60	62.5	1238	1235.4	4.6	47.2	<0.001
Summer	1620	108	78	1512	1541.9	6.6		
Autumn	1644	100	79.1	1544	1564.8	6.0		
Winter	1730	35	83.3	1695	1646.6	2.0		

Overall chi-square (χ^2^) and *p* values are presented.

**Table 3:** Observed and expected enterovirus positivity frequencies by sex.

Sex	Total	Positive (Observed)	Positive (Expected)	Negative (Observed)	Negative (Expected)	Positivity Rate (%)	χ^2^	*p*
Male	4003	193	192.7	3810	3810.2	4.8	0.001	0.976
Female	2289	110	110.2	2179	2178.7	4.8		

Overall chi-square (χ^2^) and *p* values are presented.

**Table 4:** Observed and expected enterovirus positivity frequencies by age group.

Age	Total	Positive (Observed)	Positive (Expected)	Negative (Observed)	Negative (Expected)	Positivity Rate (%)	χ^2^	*p*
Infants (0–1)	1276	131	61.4	1145	1214.5	10.2	348.5	0.001
Children (2–11)	917	123	44.1	794	872.8	13.4		
Adolescents (12–18)	162	4	7.8	158	154.1	2.4		
Adults (19–64)	1314	18	63.2	1296	1250.7	1.3		

Overall chi-square (χ^2^) and *p* values are presented.

## Data Availability

The data that support the findings of this study are available from the corresponding author on reasonable request and with appropriate authorization.

## Abbreviations

The following abbreviations are used in this manuscript:

EV	Enterovirus
COVID-19	Coronavirus disease-2019
NPI	Non-pharmaceutical intervention
SOP	Standard operating procedure
LIS	Laboratory information system
ICH	International Council for Harmonisation of Technical Requirements for Pharmaceuticals for Human Use
IQR	Interquartile range
ICU	Intensive care unit
RSV	Respiratory syncytial virus
